# The Medical Letter Bag

**Published:** 1919-04

**Authors:** 


### THE MEDICAL LETTER-BAG

A Department for Short Correspondence from Physicians

"Piece out our imperfections with your thoughts."--Shakespeare

### "The Team's the Thing."

Dear Editor:

Doctor Martin has put the matter in regard to "one man practice" so concisely that I cannot improve on it. "One man practice" should have stopped long ago. For a time one man could keep up with the increased work, but with the multiplication of essential means of diagnosis, patients must have the advantage of experts whenever necessary. Some men, of course, can do a great deal more than others, but nobody who falls short of omniscience can do everything that is required now. Think of the things needed in many cases:—serological tests, blood chemistry, ureteral catheterization, accessory sinuses, x-ray examinations from vault of cranium to sphincters. Sending specimens to laboratories is often disappointing; sending patients to separate specialists equally so. The team's the thing.

Very truly yours,

GEORGE DOCK.

### Why Diseased Teeth and Gums?

San Antonio, Texas, April 2, 1919.

My query in regard to your recent editorial, "Diseased Teeth and Diseases," was "Why diseased teeth and gums?" I had no thought of writing on the subject, but simply to get at the cause, and not the late effects. We know that diseased teeth, pyorrhoea, infected tonsils, gall bladder, appendicitis, etc., are the results of faulty nutrition. As I have expressed it for years, for lack of a better word, there is always something plus the germs. If we go back seeking an answer to our why, and try to explain along modern scientific lines as if dealing with a chemical mystery, we become confused and lost. The life giving principle, or vitamins, is lacking to a great degree in the food supplied to the masses. This does not mean the toiling, sweating masses, but to the majority of mankind. Then the saying of the eugenists, "a child has a right to be well born" is becoming or will become a necessity. While trying to agree upon a world peace and a congress of nations to prevent war in future, it seems to me something should be done to prevent the physically unfit from becoming fathers and mothers. We are spending billions each year caring for those who should never have been born. You see a chain is no stronger than its weakest link, and if we are to have good teeth we must obey that first command of God also, "Thou shalt eat bread by the sweat of thy brow." One might add, and not by the sweat of the other fellow's brow.

Very truly yours,

ROBT. E. MOSS, M. D.

---

**Pituritin in Post-Partum Hemorrhages.**

---

Texas Medical Journal:

Pituritin is a uterine stimulant increasing uterine contractions wonderfully during labor. Why would it not be one of the best remedies we could use in cases of post partum hemorrhages? I have never seen it recommended in these cases, and would like to hear from some of the profession who have used it in such cases.

Yours fraternally,

J. L. DUBOSE, M. D.,

Wells, Texas.

(Will some of our subscribers answer the inquiry made by Dr. DuBose. An exchange of opinions and experiences in the practice should be of much help to every one.—Ed.)

---

Two women of the parvenue class were discussing the future of their respective sons, when one of them said:

"Do you know, I believe that a boy's development depends largely upon his environment?"

"I know it," replied the other as she carelessly toyed with her jewel box. "There was my cousin William's boy—he never knew what it was to have a well day till the doctor found out the trouble was with his environment and cut it out."—Harper's.

